# Socio-economic determinants of mobility responses during the first wave of COVID-19 in Italy: from provinces to neighbourhoods

**DOI:** 10.1098/rsif.2021.0092

**Published:** 2021-08-04

**Authors:** Laetitia Gauvin, Paolo Bajardi, Emanuele Pepe, Brennan Lake, Filippo Privitera, Michele Tizzoni

**Affiliations:** ^1^ ISI Foundation, via Chisola, 5, 10126, Turin, Italy; ^2^ Cuebiq Inc., New York, NY, USA

**Keywords:** COVID-19, mobile phone data, human mobility

## Abstract

After more than 1 year into the COVID-19 pandemic, governments worldwide still face the challenge of adopting non-pharmaceutical interventions to mitigate the risks posed by the emergence of new SARS-CoV-2 variants and the lack of a worldwide equitable vaccine allocation. Thus, it becomes crucial to identify the drivers of mobility responses to mitigation efforts during different restriction regimes, for planning interventions that are both economically and socially sustainable while effective in controlling an outbreak. Here, using anonymous and privacy-enhanced cell phone data from Italy, we investigate the determinants of spatial variations of reductions in mobility and co-location in response to the adoption and the lift of restrictions, considering both provinces and city neighbourhoods. In large urban areas, our analysis uncovers the desertification of historic city centres, which persisted after the end of the lockdown. Such centre-periphery gradient was mainly associated with differences in educational attainment. At the province level, the local structure of the labour market mainly explained the variations in mobility responses, together with other demographic factors, such as the population’s age and sex composition. In the future, targeted interventions should take into account how the ability to comply with restrictions varies across geographical areas and socio-demographic groups.

## 1. Introduction

In the effort of fighting the COVID-19 pandemic, several governments worldwide have imposed unprecedented mobility restrictions and social distancing policies, as these—combined with contact tracing and isolation of cases—represent the most effective strategy to slow down the spread of SARS-CoV-2 [1–5] in the absence of a vaccine.

Since the very beginning of the COVID-19 pandemic, changes in human mobility ensuing the non-pharmaceutical interventions (NPIs) adopted in many countries worldwide have been measured through the analysis of mobile phone data [6]. To mention a few examples, previous studies have investigated changes in human movements through mobile phone data in Austria, China, Japan, the UK, Germany and the USA [7–10]. Several of these studies suggested that mobility restrictions unevenly impact different socio-economic strata and that income inequalities are associated with a different capacity to afford prolonged social distancing [11–13].

While it has been observed that income disparities are strongly associated with differences in mobility reductions during a lockdown, the responses to milder mitigation policies, and how such policies may impact different areas of a country depending on their local economies, have not been explored in detail. In particular, the study of mitigation policies in metropolitan areas and their impact in different neighbourhoods, remains limited to a few paradigmatic examples, like New York City [8].

As COVID-19 still poses a formidable threat to public health, due to the emergence of new variants and the lack of an equitable worldwide vaccine allocation, new measures to reduce transmission will be needed for some time and tailored solutions as an alternative to a blanket lockdown need to be developed. To understand which policies might be both sustainable and effective in controlling the outbreak, it is crucial to identify the drivers of mobility responses both during the lockdown and when restrictions are gradually lifted.

Here, we extensively investigate the socio-economic determinants of the responses to mobility restrictions imposed in Italy during the full course of the first wave of the COVID-19 epidemic, from February until June 2020, through the analysis of human mobility patterns derived from anonymized and aggregated mobile phone data. To this aim, we gauged the mobility responses during three phases of the spring COVID-19 wave in Italy: first, at the beginning of the outbreak, before the enforcement of the national lockdown; second, during the lockdown, and, third, immediately after the lift of the lockdown. We mapped mobility changes onto different spatial scales, at level of the Italian provinces and at a finer granularity, namely that of city districts in three major metropolitan areas: Turin, Milan and Rome. Mobility changes induced by self-initiated behavioural responses and by top-down interventions displayed a significant heterogeneity across all spatial scales. To investigate the socio-economic determinants that may have driven such geographical variations, we modelled their relationship with a number of demographic, economic and epidemiological covariates, including—among others—the fraction of workers by economic sector, the average personal income, the fraction of women in the population, the number of commuters and the timing of interventions.

The results show that socio-economic factors that best explain the different mobility responses by province are those related to the local labour force structure. In particular, we find a higher proportion of agriculture workers and higher levels of unemployment to be significantly associated with higher and lower levels of mobility during the lockdown, respectively. In urban areas, our results indicate that higher education levels are the strongest predictors of larger reductions in mobility in all phases of the pandemic, underscoring the unequal impact of restrictions across socio-economic strata of the population. Our findings shed light on the complex landscape of the determinants of responses to the introduction of NPIs and their relaxation.

## 2. Results

### 2.1. Spatial variations of responses to social distancing orders in Italy

To quantify the mobility responses to NPIs across Italy at the province level, we computed the average radius of gyration *r*_*g*_, a metric that captures the spatial range of users’ movements (see Methods). We evaluated *r*_*g*_ on a daily basis, averaging its value over all users who live in each of the 107 Italian provinces.

**Figure 1.**
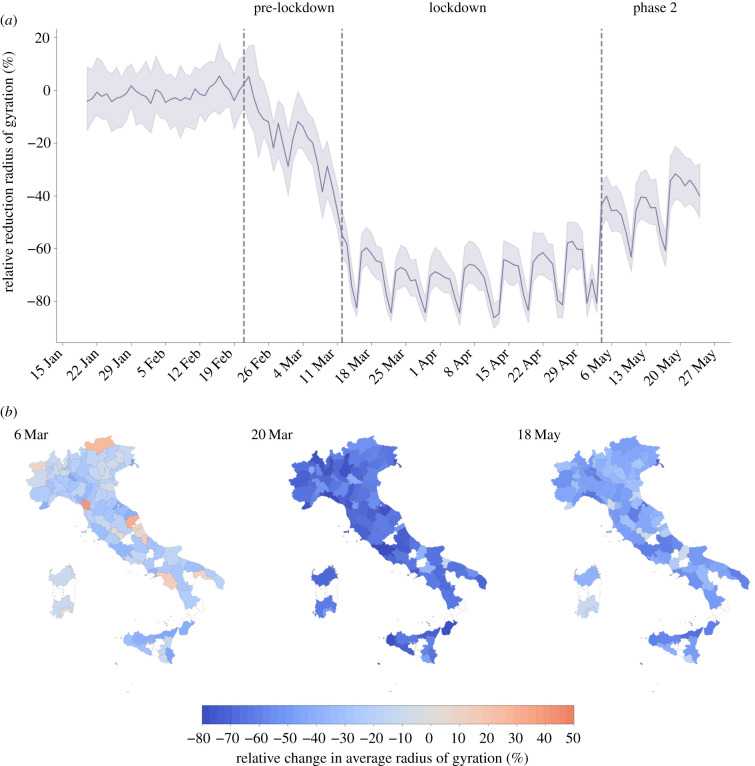
Mobility responses during the COVID-19 pandemic in Italy. Time series of the relative reduction with respect to the baseline of the median radius of gyration in Italy (*a*). The solid line indicates the median value computed across the 107 Italian provinces, while the shaded area corresponds to the 50% reference range. Dashed vertical lines mark the dates that define the three phases of the pandemic response: the pre-lockdown (between 21 February and 11 March), the lockdown (between 12 March and 4 May) and the phase 2 (after 4 May). Spatial patterns of mobility reductions (*b*). Each map shows the relative change in the average radius of gyration with respect to the baseline, by province, on 3 different days, one for each period under study. Provinces in white are not included in the analysis because of low population sampling (less than 100 users).

At the national level, the radius of gyration displayed a sharp decline with respect to its baseline value immediately after the first official report of a cluster of COVID-19 cases in Lombardy, on 21 February 2020 (figure 1). Initially, such decline was mainly due to self-induced behavioural changes, since mobility restrictions and stay-at-home orders were imposed only onto a few relatively small areas of Northern Italy. As social distancing orders became tighter, *r*_*g*_ continued to drop until a national lockdown was declared on 11 March. During the lockdown, it plateaued at an average −70% relative reduction with respect to the baseline, with distinct weekly patterns due to the movements of the active workforce employed in essential services. On 4 May, the lockdown was lifted, the so-called phase 2 started and the mobility trends reversed, although without reaching the baseline values, even after three weeks since the reopening.

Although the general mobility trend was consistent at the national level, the relative reductions of *r*_*g*_ displayed a high spatial heterogeneity. A map of the relative reductions of *r*_*g*_ (figure 1) shows that users’ mobility did not decrease uniformly across provinces during the pandemic period. For instance, on 6 March (figure 1*b*), before the lockdown, the average *r*_*g*_ had decreased by 44% in Lodi and Rimini, with respect to the baseline levels, but it had actually increased by 9% in Aosta and by 25% in Bolzano. During the lockdown, the reduction in mobility was more uniform across the country, yet showing variations between provinces. On 20 March, the average *r*_*g*_ had decreased by 80% in Rome and by 53% in Bolzano. Finally, at the start of phase 2 some provinces returned more quickly to the baseline mobility levels, while others maintained lower mobility levels for additional days and weeks. On 18 May, when all economic activities reopened, the average *r*_*g*_ had reached baseline levels in Aosta (+2%) and Sondrio (+2%) but remained at −47% with respect to the baseline in Milan.

### 2.2. Socio-economic determinants of mobility responses across the public health intervention cycle

At the level of Italian provinces, we first examined the association between the reductions in mobility and several socio-economic and epidemiological features through a mixed effects model for repeated measures for each of the three phases. Secondly, we run a multivariate regression analysis with variable selection on each day as in that way we can also extract meaningful trends [14] (see Methods for more details).

To allow a comparison of determinants, the same subset of variables are used in the mixed effects model, across the three phases (table 1). In this way, every reported result has the same interpretation: *β* represents the percentage increase/decrease in mobility given an increase of 1 s.d. in the regressor.

**Table 1.** Mixed effects models for repeated measures for the relative reductions in the median radius of gyration with respect to the baseline.

independent variables [estimates (s.e.)]	(A)	(B)	(C)
**dependent variable: relative reduction of *r*_*g*_ in pre-lockdown**			
attack rate	1.73* (0.77)	1.88* (0.81)	1.95* (0.82)
population density	1.04* (0.51)	1.51** (0.49)	1.36* (0.6)
old age index	0.72 (0.55)	—	0.97 (0.6)
high education	0.09 (0.49)	—	0.29 (0.52)
no-profit org.	−1.15* (0.57)	—	−0.90 (0.84)
unemployment	—	0.35 (0.68)	0.40 (0.84)
commuters	—	−0.56 (0.64)	−0.26 (0.67)
agriculture ratio	—	0.53 (0.57)	0.70 (0.63)
industry ratio	—	0.51 (0.66)	0.83 (0.7)
intercept	14.21*** (3.04)	14.21*** (3.02)	14.21*** (3.00)
no. of days	16	16	16
AIC	12286.70	12289.29	12291.74
**dependent variable: relative reduction of *r*_*g*_ in lockdown**			
attack rate	−0.23 (0.28)	0.61 (0.37)	1.03** (0.37)
population density	2.92*** (0.2)	2.38*** (0.18)	2.96*** (0.22)
old age index	0.69*** (0.20)	—	0.62** (0.22)
high education	0.92*** (0.18)	—	0.96*** (0.19)
no-profit org.	0.14 (0.25)	—	1.13*** (0.31)
unemployment	—	0.76* (0.31)	2.50*** (0.4)
commuters	—	−0.61* (0.24)	−0.20 (0.25)
agriculture ratio	—	−1.57*** (0.21)	−0.86*** (0.24)
industry ratio	—	−0.26 (0.25)	0.30 (0.26)
intercept	64.68*** (1.09)	64.68*** (1.07)	64.68*** (1.07)
no. of days	38	38	38
AIC	25428.28	25413.20	25367.58
**dependent variable: relative reduction of *r*_*g*_ in phase 2**			
attack rate	−1.24** (0.46)	1.31* (0.62)	1.75** (0.64)
population density	2.29*** (0.42)	2.6*** (0.38)	3.00*** (0.46)
old age index	1.21** (0.43)	—	0.80 (0.46)
high education	0.94* (0.38)	—	1.31*** (0.41)
no-profit org.	−1.99*** (0.52)	—	0.63 (0.65)
unemployment	—	2.95*** (0.66)	4.68*** (0.87)
commuters	—	−0.65 (0.49)	−0.15 (0.51)
agriculture ratio	—	−0.25 (0.48)	0.51 (0.48)
industry ratio	—	−1.26* (0.51)	−0.57 (0.53)
intercept	38.92*** (1.36)	38.92*** (1.37)	38.92*** (1.37)
no. of days	14	14	14
AIC	9952.55	9922.64	9912.82

Significance levels: *p < 0.05; **p < 0.01; ***p < 0.001.

For every period of interest, we separately run three mixed effects models: model A considers only socio-demographic variables, model B considers only economic factors and model C is the union of variables of model A and B. In every model, we always adjust by population density and attack rate. Table 1 shows model specification, coefficient estimates, and comparison across alternate specifications for robustness. According to the Akaike information criterion (AIC), including the economic factors as regressors (models B and C) leads to a preferable model for the responses in the lockdown and phase 2. Overall, in these two phases, the best model is the one that includes all the variables (model C). Model A should be preferred in the pre-lockdown period, but it is worth noting that in this phase AIC differences are small, and thus it is hard to rule out any model.

Before the national lockdown, a few variables were significantly associated with the mobility reduction: one is population density (*β* = 1.04, 95% CI [0.03, 2.04] - model A) and it remained associated with larger mobility reductions, both during the lockdown and in phase 2 (*β* = 2.96, [2.53, 3.39], and *β* = 3.00, [2.11, 3.90], respectively - model C). This means that an increase of 1 s.d. in population density led to a 1 to 3% larger reduction of *r*_*g*_ from pre-outbreak levels, suggesting that more densely populated (i.e. urban) areas were able to reduce mobility more than less densely populated provinces (i.e. rural areas).

In general, economic variables were significantly associated with mobility responses. Higher levels of unemployment were associated with a larger reduction of *r*_*g*_ during the lockdown (*β* = 2.5, [1.71, 3.3]) and after the ease of the restrictions (*β* = 4.68, [2.97, 6.39]). According to the magnitude of the coefficients, unemployment levels represented the strongest determinant of mobility reductions. For what concerns the distribution of the workforce across labour sectors, workers in the industrial and the service sectors were those most affected by the restrictions that closed all non-essential businesses, and they were encouraged to work from home once the restrictions were lifted. Indeed, provinces with a larger share of their workers employed in the industry sector experienced a larger (although not statistically significant) mobility reduction during the lockdown (*β* = 0.3, [−0.20, 0.81]). Provinces characterized by a larger agricultural workforce were characterized by a smaller reduction of *r*_*g*_, during the lockdown (*β* = −0.86, [−1.32, −0.39]). This can be expected as the entire agricultural sector remained fully active to ensure food provisioning, even during the strictest lockdown period.

From a demographic perspective, we found that an older and a more educated population was consistently positively associated with larger mobility reductions during all the three phases. The old age index was positively associated with the reductions across the three phases and was statistically significant during the national lockdown and marginally significant in phase 2 (*β* = 0.62, [0.19, 1.05] and *β* = 0.8, [−0.098, 1.70]). Higher education levels and reduction in mobility were more strongly associated, during the lockdown (*β* = 0.96, [0.58, 1.34]) and in phase 2 (*β* = 1.31, [0.51, 2.10]). Similarly, higher levels of social capital, captured by the proportion of the population who volunteer in non-profit organizations, were significantly associated with a larger reduction in mobility during the lockdown (*β* = 1.13, [0.52, 1.74]), suggesting a higher social capital leads to a higher adherence to the restrictions.

Finally, since the spread of SARS-CoV-2 in Italy was not uniform across provinces, we controlled for the local epidemic activity. The attack rate, which is the cumulative fraction of reported infections in a province up to a given date, was positively associated with larger reductions of *r*_*g*_ in every phase (*β* = 1.73, [0.21, 3.24] pre-lockdown, *β* = 1.03, [0.31, 1.76] during the lockdown, and *β* = 1.75, [0.50, 3.0] in phase 2), indicating that a higher risk perception may have led to the adoption of protective behaviours.

**Figure 2.**
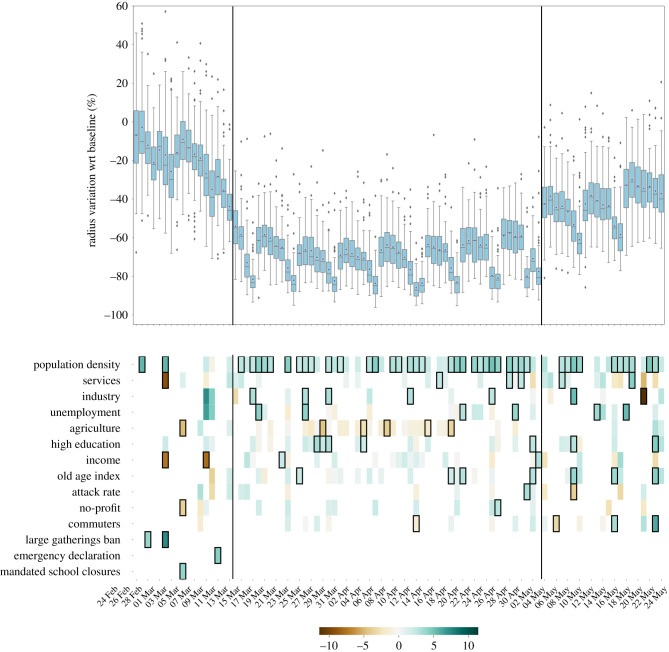
Province-level mobility reductions and their socio-economic determinants. Top panels show the relative reductions in the mean radius of gyration with respect to the baseline during the whole timeline of the dataset. Boxplots describe the distributions of the reductions by province. Bottom panels show the results of the regression model, displaying the socio-economic variables that are selected by the model to predict the corresponding variations in mobility, on each day. Coloured boxes correspond to those covariates that are selected by the model, and the colour codes the associated coefficient in the regression, from negative (brown) to positive (blue). Black solid squares highlight the covariates that are statistically significant at *p* < 0.05. Black vertical lines highlight the starting and the ending of the lockdown period.

Figure 2 shows the variables that we found to be associated with the daily reductions of *r*_*g*_, along the full period under study, through a lasso regression. Here, every day is fitted independently with a model and lasso selects a subset of variables from a larger pool (see Methods). Overall, the results are in line with those observed with the mixed effects approach. Population density was selected very often and it was always positively associated with larger mobility reductions. In figure 2, predictors are ranked according to the strength of association: the ranking highlights that four out of five top regressors are related to the labour force structure. Higher levels of unemployment were associated with a larger reduction of *r*_*g*_ in all the three phases. A higher proportion of workers in the agricultural sector was often selected with a negative *β* coefficient, indicating a higher level of mobility, while the fraction of workers in the industry sector was also positively associated with mobility reductions.

Income was seldom selected by the model as an explanatory variable and it was generally positively associated—although not significantly—with larger mobility reductions during the lockdown. Interestingly, a higher income was found to predict an increase in mobility on the Saturdays before the national stay-at-home order took effect (*β* = −7.5, [−13.8, −1.2] and *β* = −7.8, [−14.9, −0.6] on 29 February and 7 March. This result can be explained by the sudden relocation of people, out-of-home workers and students, from large cities to their place of origin, in urban belts, that followed the rumours of an imminent lockdown [15].

From a demographic perspective, we found that a more educated population was positively associated with larger mobility reductions during the three phases, while an older population was significantly positively associated only during the lockdown and phase 2. Performing an independent daily analysis, the attack rate seems to be mildly positively associated—yet not significantly—with larger reductions of *r*_*g*_, but it changes the direction of the association from time to time making it hard to draw conclusions from such behaviour. On the other hand, with this approach we can investigate more closely the effects of mandates: large gathering bans significantly reduced the average *r*_*g*_ before the lockdown, while the closures of bars and restaurants did not show a significant effect.

### 2.3. Mobility responses in the neighbourhoods of large metropolitan areas

**Figure 3.**
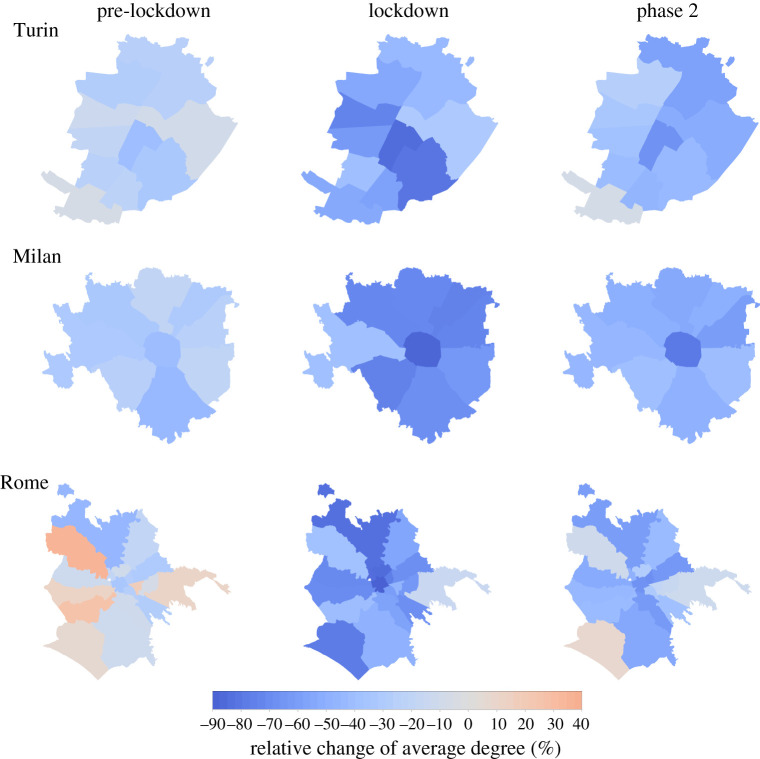
Spatial patterns of variations in co-location in metropolitan areas. Maps of the relative reduction of the average degree of the co-location network in Turin (top), Milan (middle) and Rome (bottom), by city district, on 3 different days, one for each period under study: the pre-lockdown (left), the lockdown (centre) and the phase 2 (right).

To quantify the behavioural responses to NPIs across three major Italian metropolitan areas, we computed the mean degree of the co-location network 〈*k*〉. The co-location network is built considering all the users present in a given neighbourhood every hour, and therefore it takes into account the co-presence of people who may live far away from each other (see Methods). By looking at mobility changes in urban areas, we similarly observed a high spatial variability of responses across the districts of three main Italian cities: Turin, Milan and Rome. In figure 3, the reduction of the average degree of the co-location network, 〈*k*〉, is shown for 3 days during the pre-lockdown, the lockdown and the phase 2 (corresponding to the same dates of figure 1). Turin and Milan, in the North, experienced an early decline in 〈*k*〉 before the lockdown and even in the absence of targeted measures, thus hinting to the presence of self-initiated behavioural changes. Instead, before the national lockdown the value of 〈*k*〉 had decreased with respect to the baseline only in some districts of Rome but not in others. Relative differences in social distancing decreased during the lockdown, and all cities experienced a strong reduction in 〈*k*〉, ranging between −30 and −80%, depending on the district. Overall, all the cities share a common pattern: the most central districts were those displaying the largest reduction of 〈*k*〉, since the start of the outbreak until the ease of the restrictions. Once restrictions were lifted, mobility increased more quickly in the periphery, leaving the historic city centres empty for a longer time period.

In table 2, we reported the results for three mixed effect models for repeated measures (models A, B and C) with different subsets of variables, for every phase of the study. We control for the population density in all the three models. Model A also includes socio-demographic covariates: old age index, proportion of the population with a higher education and proportion of females. Model B includes the percentage of residential buildings and the percentage of commuters. Finally, model C takes as covariates the union of the covariates of model A and model B. According to AIC, in the pre-lockdown phase, models A and C are practically equivalent, while model C is the best choice for the other phases.

**Table 2.** Random effect models for repeated measures for the relative reductions in the average degree of the co-location network with respect to the baseline.

independent variables [estimates (s.e.)]	(A)	(B)	(C)
**dependent variable: relative reduction of 〈*k*〉 in pre-lockdown**			
population density	3.33*** (0.89)	2.85*** (0.85)	3.29** (0.89)
old age index	2.43* (1.04)	—	0.74 (1.29)
high education	3.17*** (0.99)	—	3.36** (1.14)
female	−1.93 (1.11)	—	−3.21* (1.26)
commuters	—	−0.49 (0.89)	0.24 (1.08)
residential buildings	—	2.26** (0.87)	2.38* (1.08)
intercept	16.74*** (3.01)	16.74*** (3.08)	16.74*** (3.08)
no. of days	16	16	16
AIC	5429.47	5439.31	5428.65
**dependent variable: relative reduction of 〈*k*〉 in lockdown**			
population density	0.001 (0.45)	0.75 (0.47)	0.08 (0.45)
old age index	−0.10 (0.52)	—	−0.80 (0.65)
high education	9.79*** (0.5)	—	10.88*** (0.57)
female	−4.32*** (0.56)	—	−4.66*** (0.63)
commuters	—	−2.88*** (0.49)	1.98*** (0.54)
residential buildings	—	0.15 (0.48)	1.01 (0.54)
intercept	62.21*** (0.87)	62.21*** (0.87)	62.21*** (0.87)
no. of days	38	38	38
AIC	12094.05	12399.40	12082.48
**dependent variable: relative reduction of 〈*k*〉 in phase 2**			
population density	0.39 (0.83)	0.48 (0.84)	0.27 (0.82)
old age index	0.63 (0.96)	—	−1.37 (1.19)
high education	10.31*** (0.92)	—	9.8*** (1.05)
female	−5.32*** (1.03)	—	−6.98*** (1.16)
commuters	—	−4.68*** (0.88)	−1.09 (0.99)
residential buildings	—	1.34 (0.86)	2.8** (0.99)
intercept	39.34*** (1.68)	39.34*** (1.68)	39.34*** (1.68)
no. of days	14	14	14
AIC	4584.88	4665.17	4579.13

Significance levels: *p < 0.05; **p < 0.01; ***p < 0.001.

The most important feature that was associated with a larger reduction in the average degree of the co-location network, during the whole outbreak, is the proportion of people who completed a higher education degree: before the lockdown 1 s.d. increase led to an increase in the percentage reduction of the average degree of *β* = 3.17, [1.22, 5.11], and such association increased greatly during and after the lockdown (*β* = 10.88, [9.75, 12.0] and *β* = 9.8, [7.74, 11.87]). This result is intuitive, as neighbourhoods characterized by higher education levels are also the most affluent and those with a larger fraction of residents employed in professional services.

Among the other features, a higher proportion of women was negatively associated with the reduction of 〈*k*〉. The percentage of females is statistically significant with a negative coefficient during and after the lockdown (*β* = −4.66, [−5.89, −3.42] and *β* = −6.98, [−9.26, −4.71], respectively), thus indicating a higher level of co-location in places with a higher proportion of female residents. Before the lockdown the percentage of female population is always negatively associated, but it is statistically significant only when all the variables are considered (model C). After adjusting for population density, which appear to be significant only before the lockdown at this spatial resolution, a larger share of residential buildings (i.e. buildings that are not used as offices, shops or for manufacturing activities) was associated with a larger relative reduction in 〈*k*〉 before and after the lockdown.

**Figure 4.**
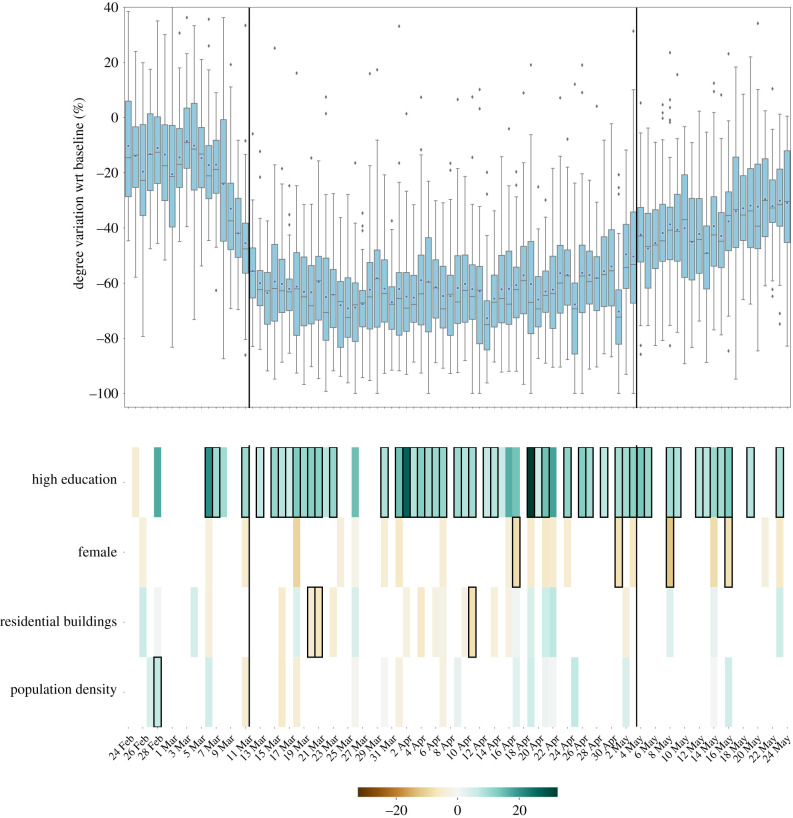
Mobility reductions in urban districts and their socio-economic determinants. Top panels show the relative reductions in the mean degree with respect to the baseline during the whole timeline of the dataset. Boxplots describe the distributions of the reductions by city district. Bottom panels show the results of the regression model, displaying the socio-economic variables that are selected by the model to predict the corresponding variations in mobility, on each day. Coloured boxes correspond to those covariates that are selected by the model, and the colour codes the associated coefficient in the regression, from negative (brown) to positive (blue). Black solid squares highlight the covariates that are statistically significant at *p* < 0.05. Black vertical lines highlight the starting and the ending of the lockdown period.

In figure 4, independent lasso models are performed on every single day. The results are consistent with the findings of the mixed effects models, but interestingly we noticed that the percentage of commuters, unemployment level and old age index are never selected as predictors. Moreover, the percentage of residential buildings appeared to be significantly negatively associated with the reduction of degree during 3 days in the national lockdown. Finally, as suggested also by the repeated measures approach, at the spatial scale of cities, the population density is rarely relevant to explain the variations in the reductions of the degree of the co-location network.

## 3. Discussion

The behavioural responses to the mitigation policies adopted by the Italian government during the first wave of the pandemic differed substantially across Italy. The spatial heterogeneity in mobility responses cannot be explained by the geographical differences observed in the spread of SARS-CoV-2 only.

Mobility reductions were observed to significantly vary both at the province level and at the city district level. In particular, in large urban areas, our data-driven analysis exposed an interesting phenomenon with several social and economic consequences: the desertification of historic city centres. As many urban centres worldwide have been severely hit by the pandemic, public health interventions prompted the relocation of residents from large cities to the countryside, as observed, for instance, in France [16] and in the USA [17]. Our analysis shows evidence of a similar effect in three major Italian cities, before and after the end of the lockdown, although, in this case, other reasons may explain the persistent divide in mobility between the centre and the periphery, such as, for instance, a higher propensity to work from home.

According to our study, the geographical differences are mainly explained by the economic factors related to the local structure of the labour force and unemployment levels, with different phases and their related mitigation policies impacting different categories of workers. According to our study, labour market factors can better capture and explain the spatial variations in mobility reductions than socio-demographic variables only.

When individual days are considered, during the lockdown mobility decreased more in provinces with a higher proportion of employees in the industry sector, as many of them were laid off with wage compensations. It also decreased more in the presence of higher unemployment, suggesting a stronger impact of the restrictions in those areas characterized by low employment levels. The share of employees in the service sector did not explain much of the variation in mobility changes before the lockdown, but it became positively associated with mobility reductions in the later period of lockdown and during the reopening. A possible explanation is that a large proportion of people working in services were either still working remotely or still not allowed to work [18]. Indeed shops, bars, restaurants and department stores were still closed on the second week of May. Moreover, remote working was widely implemented as much as possible since the early start of the outbreak and individuals whose occupation was more suitable for remote working maintained their arrangements after the end of the lockdown. Our results are in line with early observations about the disproportionate impact of the interventions due to income inequalities [19,20], and of recent studies on the effects of restrictions on local labour market areas [21].

While most of the variations in mobility are linked to the structure of the workforce share, demographic factors still played a role in the heterogeneity of the mobility responses, even though to a lesser extent. Across provinces, a higher ratio of elderly to young population was associated with a higher reduction in mobility. Previous survey-based studies have shown that non-adherence to mitigation policies in the UK was associated with a younger population, lower socio-economic grade and working in a key sector [22]. In urban areas, instead, we found a larger female population to be associated with smaller mobility reductions, in contrast to previous survey studies [23] and to previous research investigating the stay-at-home compliance during the H1N1 2009 pandemic [24]. In this case, our result may suggest responses to COVID-19 in Italy were disproportionately affecting population strata that might be more prone to comply with restrictions but could not, because financially distressed. Finally, as the outbreak was highly clustered in its early phase, we observed a strong effect of risk perception (measured by province specific attack rates) in driving the mobility reductions in Italy, as it was observed in France [16].

We acknowledge some limitations in our study. While the distribution of users resemble fairly well the distribution of the actual population both at the province level and at the district level within cities, we expect our sample to be skewed towards better educated, wealthier and younger users [25]. Mobility estimates may therefore be affected by our sample’s bias, leaning towards higher levels of mobility at baseline, before the outbreak. On the other hand, our main mobility metric, the radius of gyration, has been shown to be highly robust even in the presence of substantial biases in phone ownership and usage [26]. Moreover, anonymous digital traces do not allow investigation of and adjust for personal beliefs and people’s intrinsic motivation, including substantive moral support and social norms and expectations about how long the measures would be in place, which are known to affect intentions to comply [27,28]. Finally, our study being of an observational type, caution is needed before extrapolating direct causal effects between covariates and the observed changes in human behaviour.

In conclusion, our work underscores the important role of socio-economic factors and in particular of the labour structure [29] in shaping behavioural responses during the full course of the pandemic cycle, from early interventions to the reopening. In particular, our approach highlighted the unequal impact of mobility restrictions in urban areas, where central districts experienced a much more prolonged reduction of mobility and social contacts than the periphery. This has policy implications for the management of the pandemic in many cities worldwide, especially those characterized by large socio-economic inequalities [30]. Future intervention policies to mitigate the epidemic and hamper the economic shock, should take into account the extent to which specific population strata are able to comply. Undifferentiated restrictions might work in theory but will induce different local responses in practice because of the underlying socio-economic differences. Besides mobility limitations, adequate economic incentives [31,32] and behavioural nudging [33,34] should be implemented to maintain high levels of compliance, while additional strategies should be devised and implemented locally to protect individuals who cannot afford to comply with mobility restrictions [35–37].

## 4. Methods

### 4.1. Location data

We analyse location data provided by Cuebiq Inc. Location is collected anonymously from opted-in users, who provided access to their location through a GDPR-compliant framework. The operating system of the device (iOS or Android) combines various location data sources (e.g. GPS, WiFi networks, mobile network, beacons) to provide geographical coordinates together with an estimate of measure accuracy. Several factors may affect location accuracy (that can also vary over time for the same device), but it can be as accurate as 10 m. The data and the mobility metrics are extensively described elsewhere [38]. Here, we recall that the basic unit of information we process is an event of the form (anonymous hashed user id, time, latitude, longitude), which we call a user’s *stop* in the remainder. The duration, Δ*t*_*i*_, of a stop *i* is defined as the time elapsed between the stop *i* and the following stop *i* + 1. We perform all our analyses on a panel of about 41 000 anonymous, opted-in users for whom there was at least one stop collected during every week from 20 January 20 to 24 May. This led to about 300 million data points over the 18 weeks of this study. We average different mobility and proximity metrics during the pre-outbreak period (between 20 January and 21 February 2020) and observe their weekly and daily evolution over the course of the outbreak.

### 4.2. Definition of home location

We assign each user to a province of residence (home location) considering only their traces in the pre-outbreak period. We assume that home location is the most frequently visited nighttime location [39]. Thus we define the home province to be the province where a user has spent most of the time within the time interval 00.00–6.00, between 18 January and 21 February 2020. We consider all the stops whose duration has an intersection with the interval 00.00–06.00. For the users with home location in the provinces of Turin, Milan and Rome we further assign them to one of the city districts (or having a home out of the urban area) with the same approach. Once all users have been assigned to a home province or district, we compared their distribution with respect to official census statistics. Our users’ sample matches fairly well the distribution of the Italian population across provinces (see electronic supplementary material, figure S1) and in the districts of the three cities under study (see electronic supplementary material, figure S2).

### 4.3. Mobility metrics and mobility changes

To assess the effect of NPIs on our users’ sample, we compute two metrics that capture different notions of mobility and proximity at different scales. An extensive description of the data processing workflow to generate these metrics is reported in [38].

The first metric is an individual feature, the radius of gyration of a user *u*, *r*^*u*^_*g*_, a common measure of the spatial range covered by a user’s mobility patterns [40]. It is defined as

4.1 $$r_{g}^{u}=\frac{1}{L_{d}}\sqrt{\sum_{i=1}^{L_{d}}{\left(r_{i}-r_{\mathrm{cm}}\right)}^{2}},$$

where *L*_*d*_ is the full set of stops made by a user over a given time frame *d*, **r_i_** is the vector of coordinates of stop *i*, **r**_**cm**_ is the vector of coordinates of the centre of mass of all user’s movements, in the period *d*, weighted by the duration of each stop Δ*t*_*i*_:

4.2 $$r_{\mathrm{cm}}=\frac{1}{\sum_{i=1}^{L_{d}}\Delta t_{i}}\sum_{i=1}^{L_{d}}r_{i}\Delta t_{i}.$$

We compute the radius of gyration for each user on a daily basis, so that *L*_*d*_ in equation (4.1) represents the set of stops made by a user during a day. The radius of gyration is then averaged over all users who live in a given province.

The second metric is the average degree of a spatial co-location network that is a proxy of potential social mixing of the population. This measure is more granular and we use it as a reference metric in the analysis at the level of urban districts. To build the network, we collect all the positions of all users in a given urban district within time windows of 1 h, and we connect any two individuals with an edge if they made at least one stop within a distance *d* = 100 m from one other, within the same hour of the day. We then compute the mean hourly network degree 〈*k*〉 = 2*E*/*N*, where *E* is the number of edges and *N* is the total number of nodes in the network, including those with *k* = 0. By taking the average of 〈*k*〉 over all the 1 h slices of a given day, we obtain a daily average degree for every district.

To assess the mobility changes, we computed the reduction of the mobility metrics with respect to a baseline. The baseline values on each day of the week were obtained by averaging the mobility metrics over the five weeks before the outbreak start. The reduction of each mobility metric on a given day corresponds to the relative difference between the metrics’ value and the baseline day value. The baseline is defined independently for each spatial unit (province or neighbourhood) thus taking into account spatial variations that were present before the outbreak.

It is worth noting that the two metrics measure intrinsically different phenomena. On the one hand, the average radius of gyration of a geographical area *i* measures how far individuals move from their home place situated in *i*. On the other hand, the average degree of a geographical area *i* is related to potential mixing due to co-location events occurring in the area *i*. While from a theoretical stand point such metrics could be evaluated at any geographical resolution, we choose to study *r*_*g*_ only at the province level and 〈*k*〉 only at the city districts level. In fact, the radius of gyration of area *i* is obtained as the average of *r*_*g*_^*u*^ of users living in *i*. Thus having a large number of users is crucial to avoid biased estimates due to outlying behaviours, and city district populations were deemed too small to provide reliable estimates of it. On the contrary, regarding the average degree, large fluctuations of users sample together with heterogeneous spatial landscapes would have provided distorted estimates of 〈*k*〉, as the metric is the result of moving agents in a spatially constrained space. Thus, we choose to evaluate such metrics only on comparable areas in terms of spatial extensions and land use.

### 4.4. Explanatory variables and study design

We explore the effects of different demographic, social and economic factors on the variation of responses by gathering a range of statistical indicators from different sources. All covariates are taken from official data sources, as reported in table 3: the Italian National Institute of Statistics (ISTAT), the Ministry of Economy and Finance (MEF) and the Civil Protection Department (CPD) in charge of coordinating the national surveillance of SARS-CoV-2 (http://github.com/pcm-dpc/COVID-19). Some variables are available only at the aggregated level of provinces (e.g. number of employee of a certain productive sector), while others are collected at the finest spatial resolution of census block (e.g. number of commuters) and thus included in the analysis of city districts.

**Table 3.** Description and data source of the explanatory variables.

variable	source	province/urban	year	description
**demography**				
old age index	ISTAT	P/U	2011	ratio of people >65 years over people <15 years old
females	ISTAT	U	2011	percentage of females
social capital				
higher education	ISTAT	P/U	2011	population proportion with high school degree or higher education degree (as highest certification)
no-profit organizations	ISTAT	P	2011	population proportion volunteering in non-profit organizations
**economy**				
income	MEF	P	2018	average personal income (kEuro)
unemployment	ISTAT	P/U	2011	percentage of unemployed labour force
commuters	ISTAT	P/U	2011	percentage of people commuting daily outside the municipality of residence
agriculture	ISTAT	P	2019	percentage of employees in the agriculture sector
industry	ISTAT	P	2019	percentage of employees in the industry, manufacturing and construction sector
services	ISTAT	P	2019	percentage of employees in the services, retail, tourism sector
**territory**				
population density	ISTAT	P/U	2017	population per unit area
residential buildings	ISTAT	U	2011	percentage of buildings for dwelling purposes
COVID-19 epidemiology			
attack rate	CPD	P	2020	percentage of COVID-19 positive tests
bar/restaurant SD order	CPD	P	2020	early phase mitigation policy (binary variable)
large gathering ban	CPD	P	2020	early phase mitigation policy (binary variable)
mandatory school closure	CPD	P	2020	early phase mitigation policy (binary variable)

We consider epidemiological factors that have a direct impact on people behaviours: the presence of mandates, such as large gatherings bans or similar restrictions, and the attack rate, which is the total cumulative number of reported SARS-CoV-2 cases up to a given date, which may influence the perceived risk of being infected. These are the only indicators whose values are not constant during the time period under study. Epidemic variables were not included in the analysis of city districts because restrictions were not differentiated by neighbourhoods and because incidence data were not available at that spatial resolution.

Next, we include in our analysis demographic indicators that are expected to be associated with behavioural responses: age, described by the proportion of elderly (over 65) in the population, and gender, described by the proportion of females in the population. Previous studies have investigated the role of social capital in mediating the adherence to social distancing [41]. In our study, we account for social capital by considering two variables: the education level and the proportion of the population who volunteer in no-profit organizations. While education levels are also a proxy for income, non-profit volunteering is a more specific measure of social capital, intended as people’s propensity to cooperate, and it is often used in the literature, and especially for the case of Italy [42].

To understand the impact of the local economy [29,43], we include a number of economic variables: the average personal income, the unemployment rate, the fraction of commuters of the population, and the structure of the local workforce, described as the proportion of workers who belong to the three basic sectors: agriculture, industry and services. The latter three variables are compositional data, as they sum to the unit value, therefore in the mixed effects model, we consider only two of them (agriculture and industry) as regressors, computing their ratio with respect to the third one (services).

Finally, we control for the population density, as we expect the effects of interventions to be different between urban and rural areas. At the level of city districts, we include the proportion of residential buildings in each district, a variable that describes the gradient between the city centre, usually more residential, and the outskirts, where land use is generally more mixed.

### 4.5. Lasso linear regression and mixed effects model

To investigate possible relationships between social distancing and the socio-economic factors described above, we performed a regression analysis considering 89 provinces and the 38 districts of Milan, Rome and Turin, respectively. We include in our analysis only the provinces with a users’ sample population greater than 100 and for which all the covariates fall within 3 s.d. of their distribution.

We adopt two complementary statistical approaches, at both spatial scales: a lasso linear regression on each single day of mobility reductions, and a mixed effects model for repeated measures, with days as random effects. In both approaches, we always fit our models using standardized regressors.

The lasso regression performs variable selection [44] by minimizing the objective function given by

4.3 $$\frac{1}{2\;*\;n_{\mathrm{samples}}}\;*\;∥\Delta x_{d}-S\beta {∥}_{2}^{2}+\alpha \;*\;∥\beta {∥}_{1},$$

where *n*_samples_ is the number of data points, **x_d_** is a vector containing the reductions in mobility metric for the day *d* (either in radius of gyration or in degree) for each province (resp. urban area) and *S* is the design matrix containing the values of all the socio-economic and epidemiological covariates for each province (resp. urban area) reported in table 3. The vector *β* is a vector of the regression coefficient values. The covariates selected through the lasso regression correspond to those obtained with the best model according to cross-validation. We considered penalty parameters *α* values in the following interval [10^−3^, 10]. For the provinces, we respectively used 10 and threefolds for the cross-validation for the provinces and the cities. Once we selected the variables for each day of the three weeks considered, we run an ordinary least-squared regression using these variables.

The mixed effects model is defined by the following equation:

4.4 $$\Delta x_{i,d}=\beta_{0}+\sum_{j}\beta_{j}s_{ij}+\beta_{0d}+\epsilon ,$$

where Δ*x*_*i*,*d*_ is the reduction in mobility at day *d* for the province *i* (resp. neighbourhood), *s*_*ij*_ are the elements of the design matrix *S*, containing the standardized explanatory variables, *β*_*ij*_ are the associated fixed effects, *ε* is the error term and *β*_0*d*_ is the day random effect. We used the AIC for the optimization. In this framework, we investigated a subset of covariates, and we checked for multicollinearity based on the variance inflation factors (VIF) of all included regressors. All regressors in models A, B and C reported in Results section have VIF < 5, suggesting substantial lack of multicollinearity issues among the selected determinants.

All the analyses have been performed using the Python library *scikit-learn* for the lasso regression and the Python module *statsmodels* for the mixed effects model.

## Data accessibility

Anonymized mobility data used in this study, aggregated at the level of Italian provinces, are available at: https://doi.org/10.6084/m9.figshare.c.5029499.v2. This research also relies on data that are publicly available from the Italian National Institute of Statistics. Data sources are reported in the main text.
